# Comparative Efficacy of Ritlecitinib, Baricitinib, and Upadacitinib in Adolescents With Severe Alopecia Areata: A Bayesian Network Meta‑Analysis

**DOI:** 10.7759/cureus.117968

**Published:** 2026-10-15

**Authors:** Arya Babul, Devina Mehta, Yssra Soliman, Anand Rajpara

**Affiliations:** 1 Biomedical Sciences, West Career and Technical Academy, Las Vegas, USA; 2 NTD Elimination, Society for Awareness of Neglected Diseases, Las Vegas, USA; 3 General, Surgical, and Cosmetic Dermatology, Thomas Dermatology, Las Vegas, USA; 4 Internal Medicine, University of Nevada, Las Vegas, USA; 5 Dermatology, University of Missouri Kansas City School of Medicine, Kansas City, USA; 6 Dermatology, University Health, Kansas City, USA

**Keywords:** adolescents, alopecia areata, baricitinib, evidence synthesis, jak inhibitors, network meta-analysis, ritlecitinib, systematic review, upadacitinib

## Abstract

Severe alopecia areata (AA) in adolescents carries a substantial physical, psychological, social, and economic burden. Three Janus kinase (JAK) inhibitors, ritlecitinib, baricitinib, and upadacitinib, are now authorized in the European Union (EU) for the treatment of severe AA in both adults and adolescents, although only ritlecitinib and baricitinib are currently approved for adolescents in the United States (US). Current clinical experience in adolescents remains limited, particularly for upadacitinib and baricitinib, given their very recent introduction in the EU and US. Upadacitinib is currently under review by the US Food and Drug Administration (FDA) for adolescent AA. With multiple oral JAK inhibitors newly available or nearing approval for adolescents with severe AA, comparative evidence is needed to guide treatment selection, particularly in the context of FDA boxed safety warnings and European Medicines Agency (EMA) safety monitoring requirements. This study evaluated the relative efficacy of ritlecitinib, baricitinib, and upadacitinib in adolescents with severe AA.

A systematic review and Bayesian network meta‑analysis of randomized, placebo‑controlled trials was conducted. Adolescent data (ages 12 to 17) were extracted from eligible studies evaluating ritlecitinib, baricitinib, or upadacitinib. The primary outcome was achievement of a Severity of Alopecia Tool (SALT) score ≤20 at Week 24. Odds ratios (ORs) with 95% credible intervals (CrIs) were estimated using a fixed‑effect Bayesian model, and Week 24 SALT ≤20 responder rates were compared across trials.

Four placebo‑controlled trials were included, comprising one study each for ritlecitinib and baricitinib and two studies for upadacitinib. Data for ritlecitinib and upadacitinib were derived from predominantly adult trials with smaller adolescent subgroups, whereas data for baricitinib were derived from a dedicated pediatric study.

Compared with placebo, upadacitinib 30 mg demonstrated the highest and most stable efficacy (OR: 174.13, 95% CrI: 21.17 to 4520.81), followed by upadacitinib 15 mg (OR: 48.09, 95% CrI: 6.40 to 1304.38), baricitinib 4 mg (OR: 15.30, 95% CrI: 4.73 to 68.45), and baricitinib 2 mg (OR: 6.46, 95% CrI: 1.85 to 29.90). Indirect comparisons showed clear dose‑response relationships: upadacitinib 30 mg exceeded upadacitinib 15 mg (OR: 3.42, 95% CrI: 1.38 to 9.26), and baricitinib 4 mg exceeded baricitinib 2 mg (OR: 2.37, 95% CrI: 1.14 to 5.01). Week 24 responder rates were directionally consistent across studies: 56% to 57% and 72% to 85% of adolescents achieved SALT ≤20 with upadacitinib 15 mg and 30 mg across two studies, respectively, compared with 17% and 32% with baricitinib 2 mg and 4 mg, and 25% with ritlecitinib 50 mg. Ritlecitinib estimates were unstable because of sparse adolescent data and near‑zero placebo response rates, resulting in wide CrIs and substantial uncertainty.

Oral JAK inhibitors improved outcomes in adolescents with severe AA. Upadacitinib demonstrated a distinctly superior efficacy profile, with Week 24 responder rates far exceeding those historically observed with currently approved JAK inhibitors and markedly higher than those observed with baricitinib or ritlecitinib in this analysis. Baricitinib and ritlecitinib produced more modest and broadly similar responder rates. These findings suggest that upadacitinib may represent a higher‑efficacy tier within the JAK inhibitor class.

## Introduction and background

Alopecia areata (AA) is a chronic autoimmune disorder characterized by the collapse of hair follicle immune privilege, leading to non‑scarring hair loss affecting the scalp, eyebrows, eyelashes, and other hair‑bearing areas. The disease results from immune‑mediated destruction of hair follicles driven primarily by autoreactive T lymphocytes and inflammatory cytokine signaling pathways [1]. Although AA can occur at any age, onset during adolescence is often associated with more severe clinical manifestations, longer disease duration, and a greater psychosocial burden. Adolescents with severe AA experience increased risks of depression, anxiety, social stigma, bullying, school absenteeism, and reduced academic performance [2]. Conventional therapies, including topical corticosteroids, intralesional corticosteroids, topical immunotherapy, and systemic immunosuppressants, frequently demonstrate variable efficacy and high relapse rates [3].

The introduction of Janus kinase (JAK) inhibitors to treat AA has transformed the management of severe AA and ushered in a modern paradigm in clinical trial methodology, including optimized study design and outcomes assessment [4-12]. However, a substantial unmet need remains for therapies that are more effective, safer, and capable of providing more durable disease control.

Pivotal clinical trials consistently show that a substantial proportion of treated patients do not achieve clinically meaningful scalp hair regrowth, defined as a Severity of Alopecia Tool (SALT) score ≤20, after extended treatment durations of up to 52 weeks [4-14]. Durability of response also remains a challenge. Among patients who initially experience significant improvement, interruption of baricitinib therapy has been associated with early decline in hair regrowth for some individuals and marked loss of benefit during longer periods of follow-up, with only partial recovery of response when treatment is resumed (2 mg, 63%; 4 mg, 85%) [7]. A comparable pattern has been observed with ritlecitinib, where retreatment restores improvement in only 53% of prior responders, defined as ≥30% improvement in SALT score from baseline [6,14]. Given these limitations, and in the absence of a new, mechanistically differentiated pharmacologic class with an approved indication for severe AA, suboptimal efficacy or tolerability will likely necessitate switching among available oral JAK inhibitors to leverage interindividual or intraclass differences, as suggested in recent reports [15-21].

Baricitinib, ritlecitinib, and deuruxolitinib were commercialized in the United States (US) for severe AA in 2022, 2023, and 2025, respectively [13,22,23]. Among these agents, ritlecitinib and baricitinib are now approved in the US for adolescents with severe AA. Dermatologists have accumulated several years of clinical experience with ritlecitinib in adolescents across major regulatory markets, whereas baricitinib has only recently become available for this population in both the European Union (EU) and the US [24,25]. Upadacitinib has been authorized in the EU for severe AA in both adults and adolescents and remains under review by the US Food and Drug Administration (FDA) for this indication [26].

Baricitinib is a selective JAK1 and JAK2 inhibitor that suppresses inflammatory cytokine signaling pathways implicated in AA pathogenesis, particularly interferon-gamma-mediated immune activation [11,22,27]. Ritlecitinib is a selective JAK3 and TEC (tyrosine kinase expressed in hepatocellular carcinoma) family kinase inhibitor that reduces lymphocyte activation and immune‑mediated follicular inflammation [14,23,28,29]. Upadacitinib has been approved in major regulatory markets for several inflammatory conditions such as atopic dermatitis and rheumatoid arthritis, and recent evidence demonstrates robust efficacy in adults and adolescents with severe AA. Its selective inhibition of JAK1 reduces proinflammatory cytokine signaling relevant to AA pathogenesis [30]. It also reduces downstream activation of STAT3 and STAT5 in response to cytokines such as interleukin-6 and interleukin-7, reflecting targeted suppression of JAK1-mediated signaling pathways. This pattern of cytokine modulation is relevant in AA because attenuation of gamma chain cytokine activity, including interleukin-15 and interleukin-7, limits the activation and persistence of cytotoxic T cells that contribute to follicular inflammation [31]. 

Despite these advances, no direct comparisons among ritlecitinib, baricitinib, and upadacitinib in adolescents with severe AA exist, and importantly, no indirect comparisons such as a network meta‑analysis (NMA) have been conducted to date. Most available trials evaluate individual agents against placebo, which limits the ability to determine the relative efficacy and safety of these therapies [11,12,24,32]. Evidence regarding eyebrow and eyelash regrowth in adolescents is also limited.

Real‑world prescribing trends presented at a recent American Academy of Dermatology meeting indicate that clinicians often initiate JAK inhibitors at lower doses and are hesitant to escalate to higher doses due to multiple boxed warnings, the need for ongoing laboratory monitoring, adverse events such as acne, and patient reluctance [33]. Such caution is likely even more pronounced in adolescents. Against this backdrop, given that approximately 75% of adolescents treated with ritlecitinib 50 mg did not achieve clinically meaningful scalp hair regrowth at 24 weeks and approximately 50% had not done so by 48 weeks [12], dermatologists may be well motivated to extend their clinical experience with baricitinib, now recently approved for severe AA in both the EU and US, and with upadacitinib, recently approved in the EU and under active regulatory review by the FDA for this indication.

NMA allows indirect comparison of multiple therapies when direct comparative trials are lacking. By integrating evidence from placebo‑controlled trials, NMA enables estimation of comparative treatment effects within a connected evidence network. A Bayesian framework allows probabilistic interpretation of treatment effects and accommodates sparse adolescent subgroup data more flexibly than frequentist approaches. Therefore, the present study aimed to perform a systematic review and Bayesian NMA evaluating the comparative efficacy of oral JAK inhibitors used for severe AA in adolescents, specifically ritlecitinib, baricitinib, and upadacitinib. All three agents are authorized by the European Medicines Agency (EMA) for adolescents with severe AA, with ritlecitinib and baricitinib now approved in the US and upadacitinib remaining under review by the FDA. The primary outcome was achievement of a SALT score ≤20 at Week 24. Secondary outcomes included eyebrow response and eyelash response.

## Review

Methods

Study Design

An exploratory placebo-anchored indirect comparison and Bayesian NMA [34] was conducted to compare the efficacy and safety of JAK inhibitors in adolescents with severe AA. The study followed Preferred Reporting Items for Systematic Reviews and Meta-Analyses (PRISMA)-NMA recommendations [35] and was conducted in accordance with an a priori protocol registered in PROSPERO (CRD420251162672).

Literature Search

A comprehensive literature search was conducted from database inception to August 14, 2026, in PubMed, Embase, CENTRAL, and ClinicalTrials.gov. Controlled vocabulary and free‑text terms were combined for alopecia areata, ritlecitinib, baricitinib, upadacitinib, deuruxolitinib, ivarmacitinib, rezpegaldesleukin, and randomized controlled trials. The search was supplemented with data from U.S. Prescribing Information and the EMA Summary of Product Characteristics (SmPC).

Eligibility Criteria

Included studies met the following criteria: adolescents with severe AA (baseline SALT score ≥50); assessment of ritlecitinib, baricitinib, or upadacitinib and a placebo arm; data from randomized placebo‑controlled clinical trials; use of oral JAK inhibitors, approved or under regulatory review for adolescents; monotherapy administration; and availability of Week 24 SALT score of ≤20 outcomes. In line with FDA guidelines, adolescents refer to children aged 12 to 17 years [36]. This age range is also applied in FDA‑approved prescribing information to structure adolescent labeling and safety and efficacy analyses. 

Studies were excluded if they were non‑randomized, unblinded, not prospective, observational, involved combination therapy, were conference abstracts lacking sufficient extractable data, were duplicates, lacked adolescent‑specific outcomes, or were not published in English.

Treatment arms included baricitinib 2 mg and 4 mg; ritlecitinib 30 mg, 50 mg, 200/30 mg, and 200/50 mg; and upadacitinib 15 mg and 30 mg. The ritlecitinib 200 mg regimens consisted of a 4‑week loading dose followed by daily maintenance dosing. Although only the 50 mg daily dose without a loading dose was eventually approved, the FDA new drug application review noted that all four ritlecitinib regimens demonstrated clinically meaningful achievement of SALT scores ≤20 [37]. The ritlecitinib 10-mg arm was excluded because it was designed primarily for pharmacokinetic evaluation.

Outcomes

The primary endpoint was achievement of a SALT score ≤20 at Week 24, based on extractable binary efficacy outcomes. Secondary exploratory outcomes included eyebrow and eyelash responses at Week 24 or the nearest comparable time point. Because of heterogeneity in definitions, assessment methods, and across adolescent datasets, formal NMA for eyebrow and eyelash was not appropriate. Eyebrow and eyelash outcomes were therefore summarized descriptively using reported responder proportions.

Data Extraction

Responder counts and total sample sizes were extracted from each treatment arm. When only percentages were reported, event counts were calculated using the reported denominators. Extracted variables included study characteristics, intervention regimens, sample sizes, efficacy outcomes, and safety outcomes. Placebo arms served as the common comparator for the evidence network.

Statistical Analysis

A fixed-effect Bayesian NMA was performed because of the limited number of adolescent studies and small sample sizes. Odds ratios (ORs) with 95% credible intervals (CrIs) were calculated for all indirect pairwise comparisons [38]. The primary comparative analysis focused on the Week 24 SALT score of ≤20 response rates. For secondary outcomes lacking consistency in the timing of data collection, descriptive analysis was carried out. The analysis was performed using R software (R Foundation for Statistical Computing, Vienna, Austria). The results obtained were a league table of indirect comparisons and forest plots of the ORs relative to placebo. Heterogeneity was measured using the I² statistic, and model fit with residual deviance and deviance information criterion (DIC).

Risk of Bias and Publication Bias

Risk of bias was assessed using the Cochrane Risk of Bias 2 (RoB 2) tool [39]. Publication bias and small-study effects were explored visually using comparison-adjusted funnel plots, although interpretation was limited by the small number of included studies.

Results

Study Selection

The PRISMA-based systematic literature search (Figure 1) identified 82 records. After removal of 42 duplicate records, 40 studies underwent screening and 29 were excluded. Eleven reports were assessed for full-text eligibility, and seven were excluded because of non-randomized designs, non-AA populations, absence of placebo comparators, or conference abstract-only publications without sufficient extractable adolescent‑specific data. Ultimately, four randomized placebo-controlled clinical trials were eligible for inclusion in the final Bayesian NMA: one ritlecitinib trial (ALLEGRO phase 2b/3; NCT03732807), one baricitinib trial (BRAVE-AA-PEDS; NCT05723198), and two upadacitinib phase 3 clinical trials (UP-AA; NCT06012240) [12,31,40]. The EMA SmPC also provides separate efficacy data for severe AA in adolescents [24,30]. 

**Figure 1:**
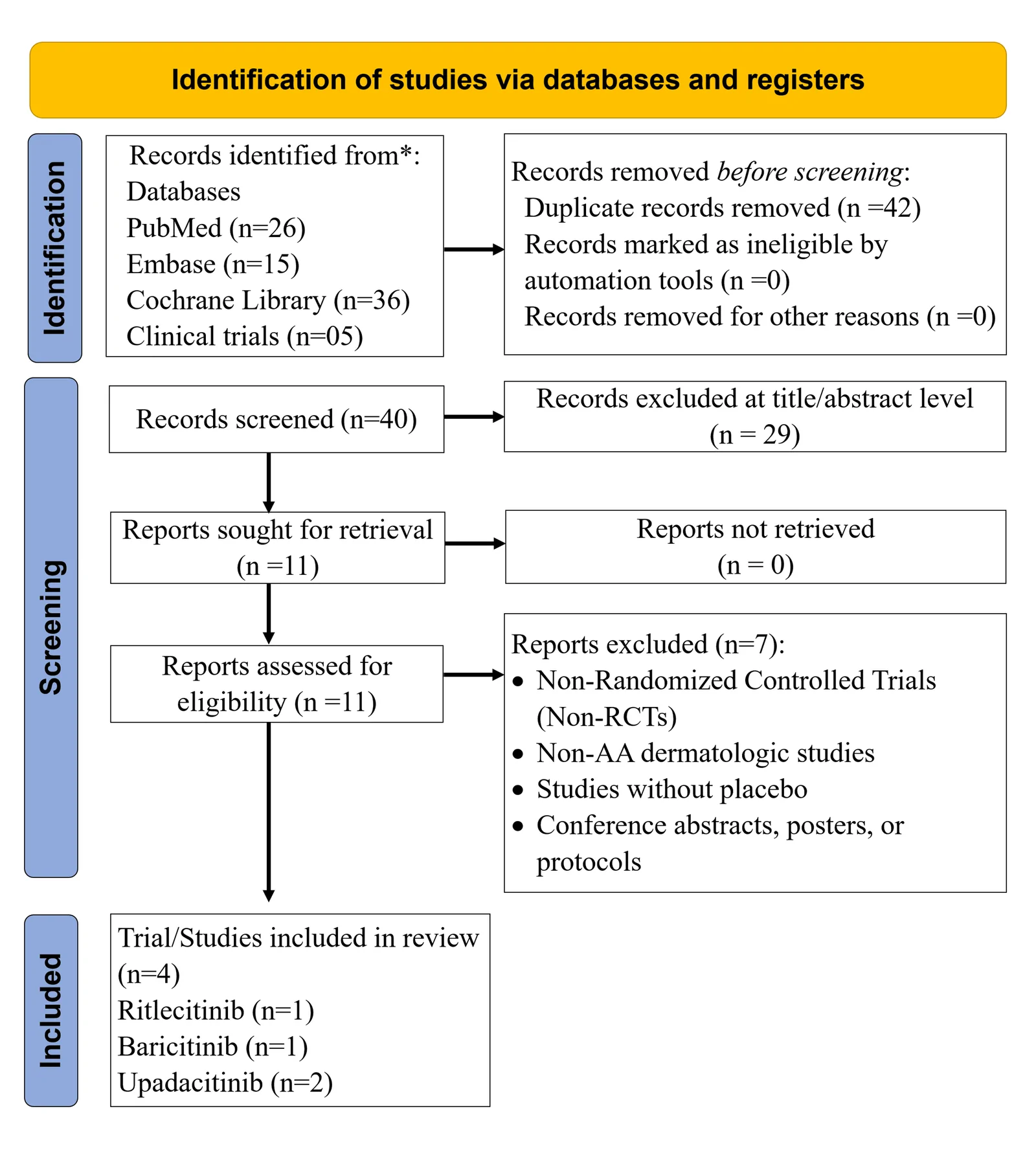
PRISMA Flow Diagram of Study Selection Sources: Hordinsky et al., 2023, ritlecitinib [12]; Passeron et al., 2025, baricitinib [40]; and Mostaghimi et al., 2026, upadacitinib (Studies 1 and 2 in one report) [31]. AA, alopecia areata; PRISMA: Preferred Reporting Items for Systematic Reviews and Meta-Analyses; RCT, randomized controlled trial.

Study Characteristics

Detailed study characteristics are provided in Table 1. All included studies were international, multicenter randomized placebo-controlled trials. Evaluated interventions included ritlecitinib 30 mg, 50 mg, 200/30 mg, and 200/50 mg; baricitinib 2 mg and 4 mg; and upadacitinib 15 mg and 30 mg, dosed once daily. Sample sizes varied across treatment arms, with the largest adolescent population in the dedicated BRAVE-AA-PEDS pediatric baricitinib study [40], while ritlecitinib ALLEGRO [12] and upadacitinib UP-AA [31] data were derived from predominantly adult trials with smaller adolescent subgroups.

**Table 1:** Characteristics of Included Clinical Trials in Adolescents The Mostaghimi et al., 2026 report [31] comprises two randomized, placebo‑controlled trials of updacitinib. Responders (n) represent participants achieving a SALT score of ≤20 at Week 24; SALT score of ≤20 (w/o placebo) represents the placebo-adjusted response rate, calculated by subtracting the placebo response within the corresponding trial (or within the corresponding study for UP-AA Study 1 and Study 2) from the observed active treatment response; percentages are rounded; minor differences may be due to rounding. NCT, National Clinical Trial identifier; QD, once daily; SALT, Severity of Alopecia Tool; S1, Study 1; S2, Study 2.

Author, year, NCT, reference	Trial name	Design	Setting	Intervention arms	Sample size (per arm)	Responders (n)	SALT score ≤20	SALT score ≤20 (w/o placebo)
Hordinsky et al., 2023 (NCT03732807) [12]	ALLEGRO phase 2b/3	International, randomized, double-blind, placebo-controlled, combined dose-ranging pivotal phase 2b/3 trial	118 sites in Argentina, Australia, Canada, Chile, China, Colombia, Czech Republic, Germany, Hungary, Japan, Korea, Mexico, Poland, Russia, Spain, Taiwan, the UK, and the USA	Placebo	19	0	0.0	N/A
				Ritlecitinib 200/50 mg	18	5	27.8	27.78
				Ritlecitinib 200/30 mg	17	3	17.6	17.65
				Ritlecitinib 50 mg	16	3	18.8	25
				Ritlecitinib 30 mg	18	3	16.7	16.67
Passeron et al., 2025 (NCT05723198) [40]	BRAVE-AA-PEDS	International, randomized, double-blind, placebo-controlled, combined dose-ranging phase 3 pivotal trial	140 sites in Argentina, Australia, Canada, France, Germany, Hungary, Japan, Mexico, Poland, South Korea, Spain, Taiwan, and the USA	Placebo	88	3	3.4	
				Baricitinib 2 mg	84	14	16.7	13.26
				Baricitinib 4 mg	85	27	31.8	28.35
Mostaghimi et al., 2026 (NCT06012240) [31]	UP-AA (Study 1 and Study 2)	International, randomized, double-blind, placebo-controlled, combined dose-ranging pivotal phase 3 replicate trials	269 sites in Argentina, Australia, Belgium, Brazil, Bulgaria, Canada, Chile, China, Croatia, France, Germany, Greece, Hungary, Israel, Italy, Japan, New Zealand, Poland, Portugal, Puerto Rico, Slovakia, South Korea, Spain, Sweden, Taiwan, Turkey, and the USA	Placebo	13	0	0.0	N/A
				Upadacitinib 15 mg QD (UPA15) (S1)	25	14	56.0	56
				Upadacitinib 30 mg QD (UPA30) (S1)	26	22	84.6	54.62
				Placebo	10	1	10.0	
				Upadacitinib 15 mg QD (UPA15) (S2)	23	13	56.5	46.52
				Upadacitinib 30 mg QD (UPA30) (S2)	21	16	76.2	66.19

Network Geometry

The evidence network included placebo-controlled comparisons among ritlecitinib, baricitinib, and upadacitinib regimens evaluated for achievement of a SALT score ≤20 in adolescents with severe AA (Figure 2). Network plots demonstrated adequate connectivity for indirect treatment comparisons, although several treatment arms were informed by sparse subgroup data.

**Figure 2:**
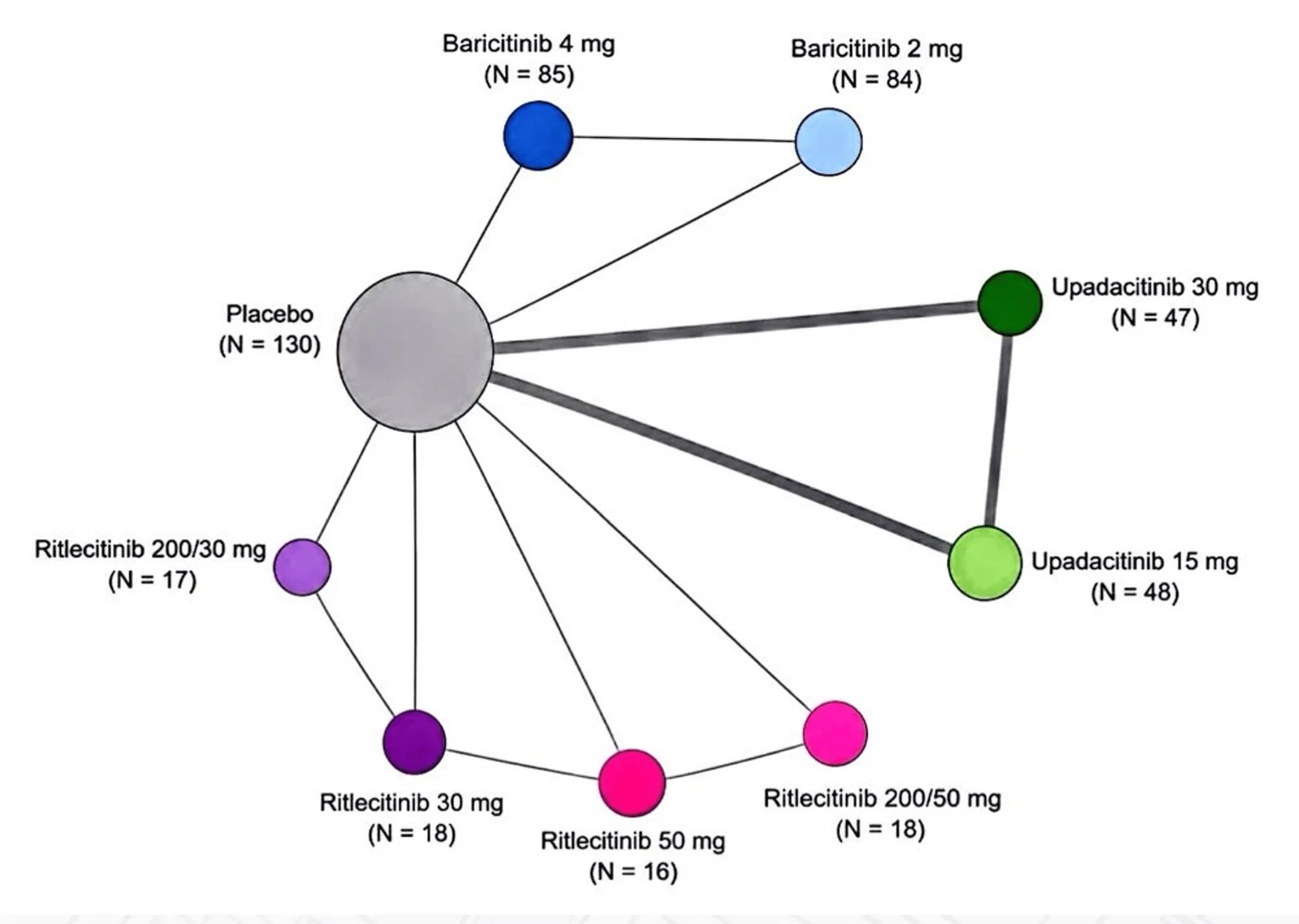
Network Geometry for the Bayesian Network Meta-Analysis Network plot of placebo‑controlled comparisons of baricitinib, ritlecitinib, and upadacitinib in adolescents. Node size reflects sample size, and line thickness reflects direct comparisons. Placebo was the common comparator. Sources: Hordinsky et al., 2023 (ritlecitinib [12]); Passeron et al., 2025 (baricitinib [40]); Mostaghimi et al., 2026 (upadacitinib [31]).

Bayesian NMA

A Bayesian fixed-effect NMA was conducted because statistical heterogeneity across the network was low (I² = 6%). Model fit was acceptable, with residual deviance closely approximating the number of unconstrained data points (residual deviance ratio: 0.99) (Figure 3).

**Figure 3:**
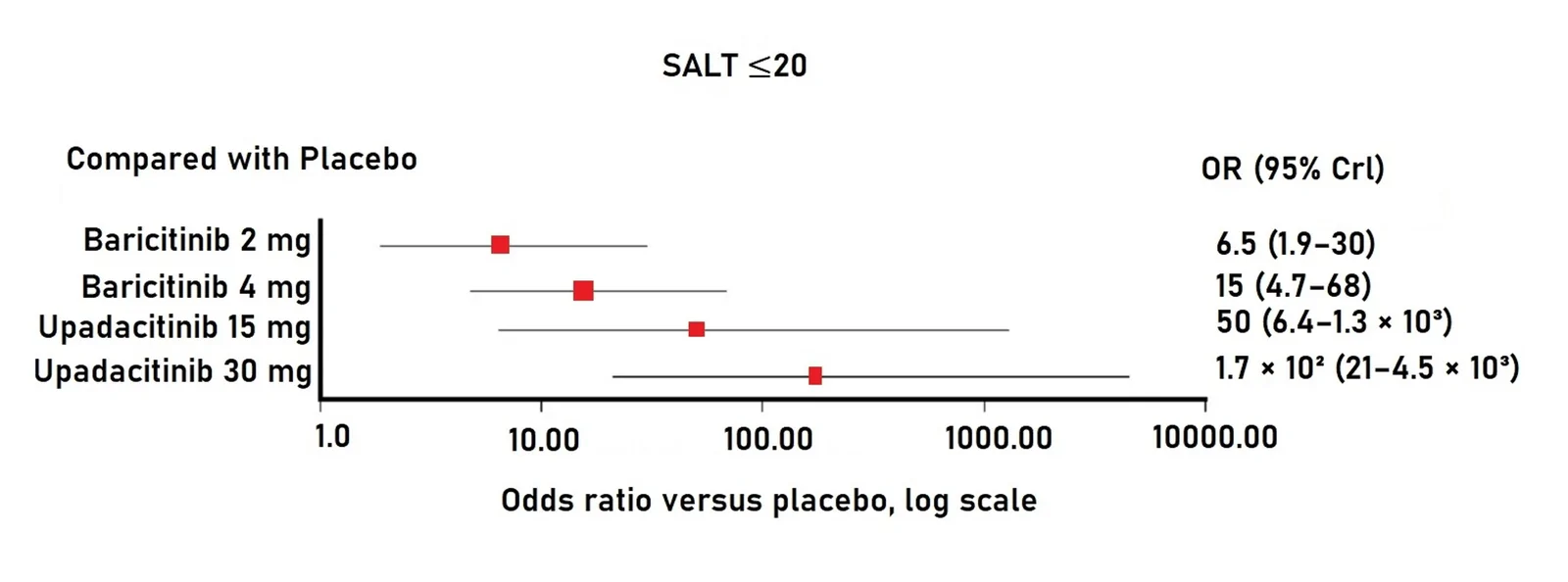
Forest Plot of Treatment Effects Versus Placebo Odds ratios (ORs) with 95% credible intervals (CrIs) from the fixed-effect Bayesian network meta-analysis. Values greater than 1 favor active treatment versus placebo. Upadacitinib 30 mg demonstrated the highest and most stable efficacy estimate. Comparisons with ritlecitinib exhibited substantial statistical uncertainty because of sparse adolescent subgroup data and near-zero placebo response rates. Ritlecitinib treatment arms were omitted from Figure 3 for clarity because sparse-event data produced unstable and implausibly large estimates. Sources: Passeron et al., 2025 (baricitinib [40]); Mostaghimi et al., 2026 (upadacitinib [31]). SALT, Severity of Alopecia Tool.

Compared with placebo, all JAK inhibitor regimens increased the likelihood of achieving a SALT score of ≤20. Upadacitinib 30 mg demonstrated the highest and most stable efficacy estimates versus placebo (OR: 174.13, 95% CrI: 21.17 to 4520.81), followed by upadacitinib 15 mg (OR: 48.09, 95% CrI: 6.40 to 1304.38). Baricitinib 4 mg demonstrated greater efficacy compared with baricitinib 2 mg, with ORs versus placebo of 15.30 (95% CrI: 4.73 to 68.45) and 6.46 (95% CrI: 1.85 to 29.90), respectively.

Ritlecitinib regimens produced extremely large effect estimates with very wide CrIs. For example, ritlecitinib 50 mg demonstrated an OR versus placebo exceeding 10¹⁰, reflecting the instability introduced by sparse adolescent data and near‑zero placebo responses.

Indirect Comparisons

Indirect comparisons showed a dose‑response for baricitinib and upadacitinib (Table 2). Upadacitinib 30 mg was more effective than 15 mg (OR: 3.42, 95% CrI: 1.38 to 9.26), and baricitinib 4 mg was more effective than 2 mg (OR: 2.37, 95% CrI: 1.14 to 5.01). Ritlecitinib estimates were unstable due to sparse data and zero‑event placebo arms.

**Table 2:** Indirect Comparisons of Oral JAK Inhibitors for the Achievement of SALT Score ≤20 in Adolescents With Severe Alopecia Areata: Bayesian Network Meta-Analysis Results Data are presented as odds ratios (ORs) with 95% credible intervals (CrIs) from the Bayesian network meta‑analysis. An OR >1 favors the treatment listed in the row over the treatment listed in the column. Comparisons with 95% CrIs excluding 1 are considered statistically significant. Extremely large ORs and very wide CrIs (or values reported as 0 due to rounding) reflect sparse‑event data and should be interpreted with caution. For consistency, very large point estimates were converted to scientific notation. JAK, Janus kinase; N/A, not applicable; SALT, Severity of Alopecia Tool.

Treatment	Baricitinib 2 mg	Baricitinib 4 mg	Ritlecitinib 30 mg	Ritlecitinib 50 mg	Ritlecitinib 200/30 mg	Ritlecitinib 200/50 mg	Upadacitinib 15 mg	Upadacitinib 30 mg
Baricitinib 2 mg	N/A	N/A	N/A	N/A	N/A	N/A	N/A	N/A
Baricitinib 4 mg	2.37 (1.14-5.01)	N/A	N/A	N/A	N/A	N/A	N/A	N/A
Ritlecitinib 30 mg	2.31E+8 (1.44-3.18E+24)	9.90E+7 (0.61-1.48E+24)	N/A	N/A	N/A	N/A	N/A	N/A
Ritlecitinib 50 mg	3.92E+8 (2.55-5.59E+24)	1.68E+8 (1.04-2.26E+24)	1.72 (0.30-11.70)	N/A	N/A	N/A	N/A	N/A
Ritlecitinib 200/30 mg	2.42E+8 (1.57-3.57E+24)	1.05E+8 (0.65-1.45E+24)	1.08 (0.16-7.29)	0.63 (0.10-3.62)	N/A	N/A	N/A	N/A
Ritlecitinib 200/50 mg	4.69E+8 (3.03-6.76E+24)	1.99E+8 (1.28-2.79E+24)	2.02 (0.39-12.80)	1.17 (0.24-6.14)	1.88 (0.36-11.57)	N/A	N/A	N/A
Upadacitinib 15 mg	6.76 (0.55-256.97)	2.85 (0.24-106.81)	0 (0-5.62)	0 (0-3.39)	0 (0-5.44)	0 (0-2.73)	N/A	N/A
Upadacitinib 30 mg	23.38 (1.89-963.01)	9.90 (0.81-378.46)	0 (0-19.49)	0 (0-11.06)	0 (0-18.16)	0 (0-9.37)	3.42 (1.38-9.26)	N/A

Observed Week 24 SALT ≤20 Responder Rates Across Trials

Direct responder rates provided additional clarity (Figure 4). In the ritlecitinib study, 25% of adolescents receiving the approved 50-mg dose achieved SALT score ≤20, indicating that 75% did not achieve clinically meaningful scalp hair regrowth by Week 24 [12]. In the baricitinib study, 16.6% and 31.8% of adolescents receiving baricitinib 2 mg and 4 mg achieved SALT score of ≤20, corresponding to non-response rates of 83.4% and 68.2% [40].

**Figure 4:**
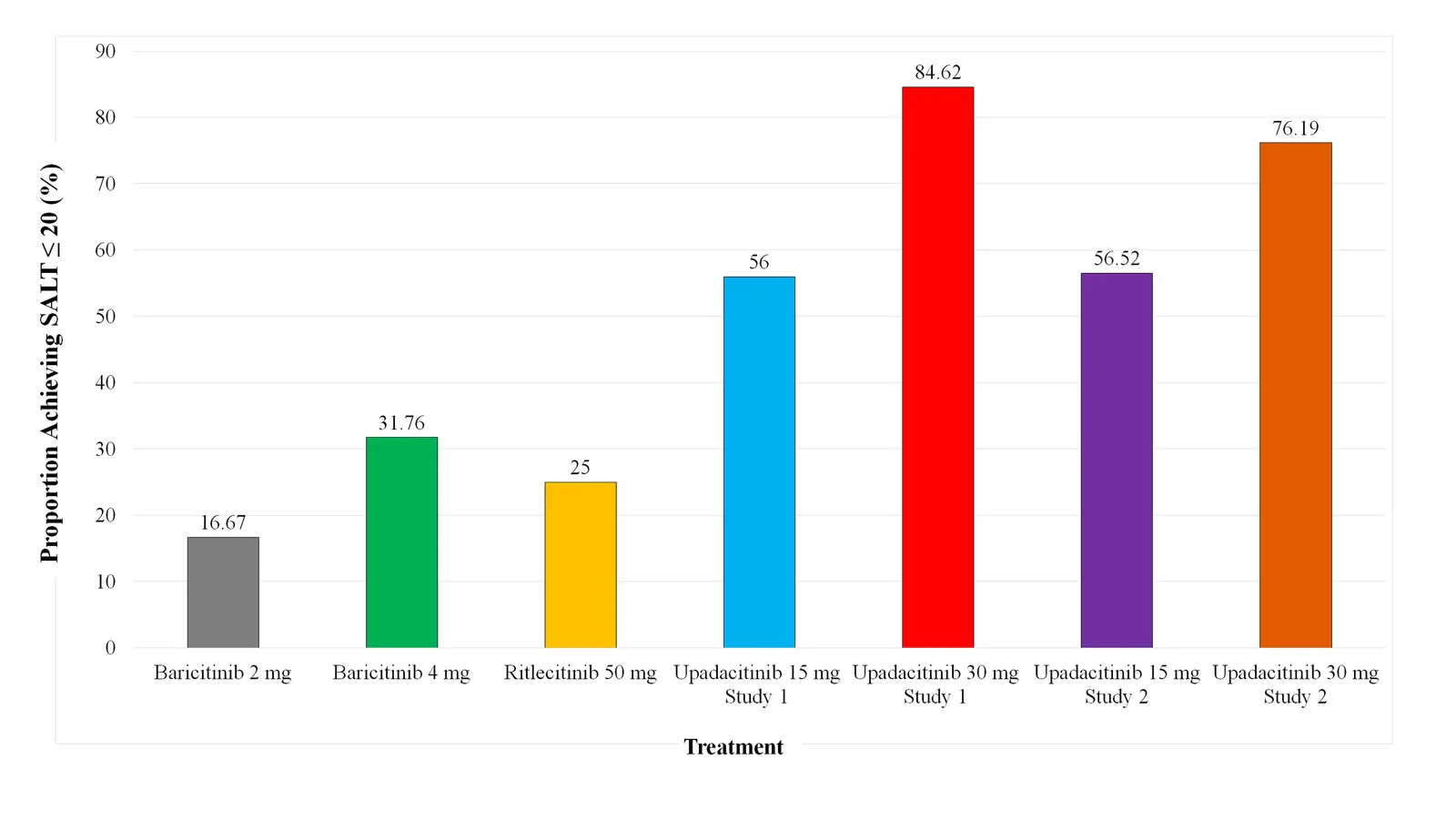
Meaningful Scalp Hair Regrowth by Week 24 (SALT ≤20) Proportions of adolescents with severe AA achieving a SALT score of ≤20 at Week 24 with baricitinib 2 mg and 4 mg, ritlecitinib 50 mg, and upadacitinib 15 mg and 30 mg (Studies 1 and 2). Week 24 response rates were 16.6% and 31.8% for baricitinib, 25% for ritlecitinib, 56.0% and 56.5% for upadacitinib 15 mg, and 84.6% and 72.2% for upadacitinib 30 mg. Corresponding non‑response rates (SALT >21) were 83.4%, 68.2%, 75%, 44% and 43.5%, and 15.4% and 27.8% for baricitinib 2 mg, baricitinib 4 mg, ritlecitinib 50 mg, upadacitinib 15 mg, and upadacitinib 30 mg, respectively, highlighting the numerically higher responder rates with upadacitinib. Sources: Hordinsky et al., 2023 (ritlecitinib [12]); Passeron et al., 2025 (baricitinib [40]); Mostaghimi et al., 2026 (upadacitinib [31]). AA, alopecia areata; SALT, Severity of Alopecia Tool.

In the UP-AA trials at Week 24, 56.0% and 56.5% of adolescents receiving upadacitinib 15 mg, and 84.6% and 72.2% receiving upadacitinib 30 mg achieved SALT score of ≤20. These findings correspond to non-response rates of approximately 44% and 43.5% on upadacitinib 15 mg, and 15.4% and 27.8% on upadacitinib 30 mg [31,32]. 

The response rates observed among all the JAK inhibitors were consistently aligned with the Bayesian NMA and indirect comparisons. The best efficacy was observed for upadacitinib 15 and 30 mg, whereas the responses were more modest and comparable in case of baricitinib and ritlecitinib. However, even with the use of a 4-week loading dose of 200 mg followed by maintenance dose of 50 mg (200/50 mg), the response rate for ritlecitinib at week 24 was 27.8%, closely approximating the response observed with baricitinib 4 mg.

Eyelash and Eyebrow Response Outcomes

Adolescent eyelash and eyebrow responses were higher for all JAK inhibitor groups than placebo (Figure 5). Upadacitinib 30 mg had the highest rates (80.8% and 92.9%), with upadacitinib 15 mg showing intermediate responses (~35%). Ritlecitinib 200/50 mg produced responses of ~61.5% and 40%, while the approved 50-mg dose yielded ~20% and 40%. Placebo responses were minimal. Eyelash and eyebrow efficacy generally mirrored primary outcomes.

**Figure 5:**
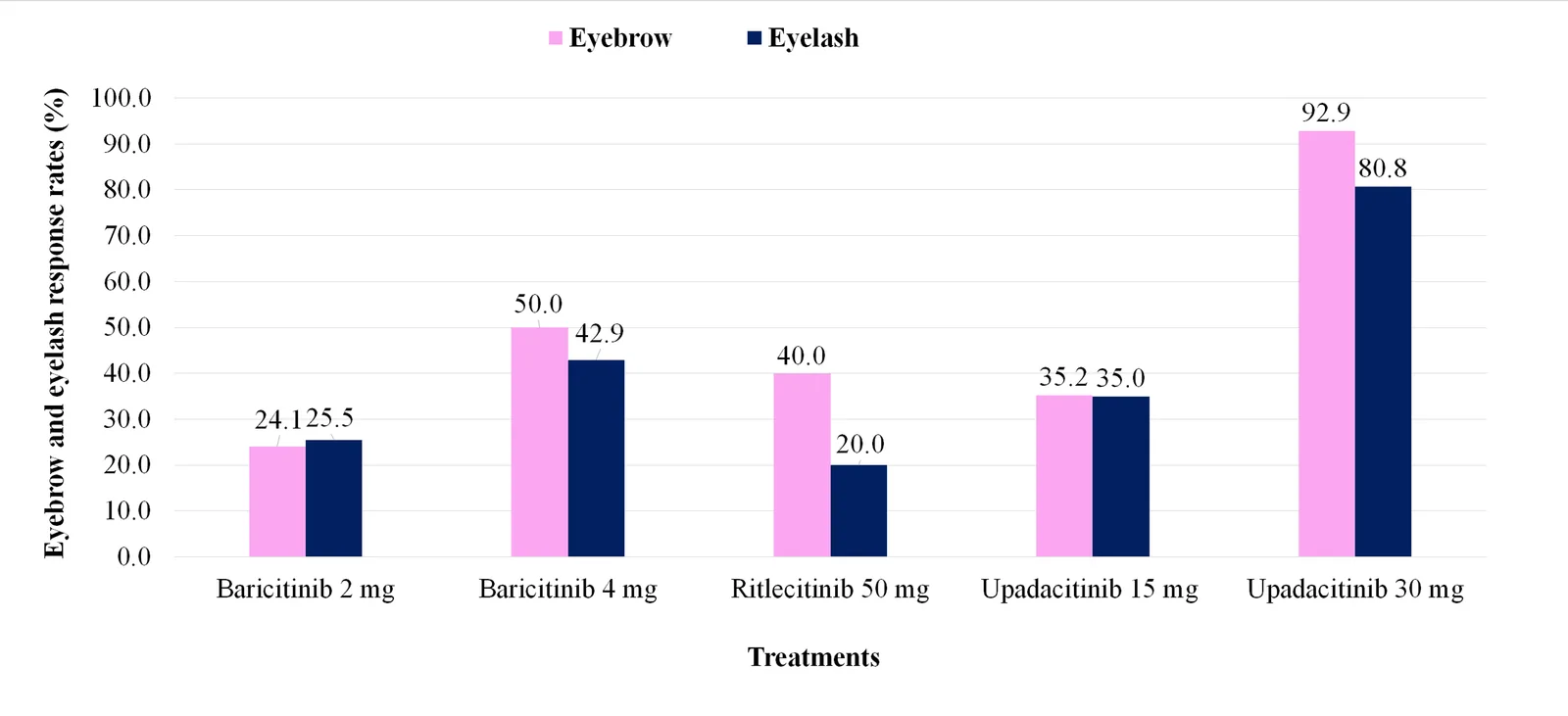
Eyebrow and Eyelash Response Rates Among Adolescents Receiving JAK Inhibitors Eyebrow and eyelash response rates in adolescents with severe AA treated with baricitinib 2 mg and 4 mg, ritlecitinib 50 mg, and upadacitinib 15 mg and 30 mg (two‑study aggregate). Eyebrow responses were 24.11%, 50%, 40%, 35.2%, and 92.9%; eyelash responses were 25.5%, 42.9%, 20%, 35%, and 80.8%. Response required a ClinRO score of 0-1 plus a ≥2‑point improvement. ClinRO scales are 4‑point measures (0-3). Upadacitinib 30 mg showed the highest rates. No NMA was performed due to heterogeneity. Sources: Hordinsky et al., 2023 (ritlecitinib [12]); Passeron et al., 2025 (baricitinib [40]); Mostaghimi et al., 2026 (upadacitinib [31]). JAK, Janus kinase; NMA, network meta‑analysis.

Publication Bias and Small-Study Effects

Visual inspection of the comparison‑adjusted funnel plot (Figure 6) did not indicate major asymmetry, though interpretation is limited by the small number of studies, sparse adolescent subgroup data, and several rare‑event comparisons.

**Figure 6:**
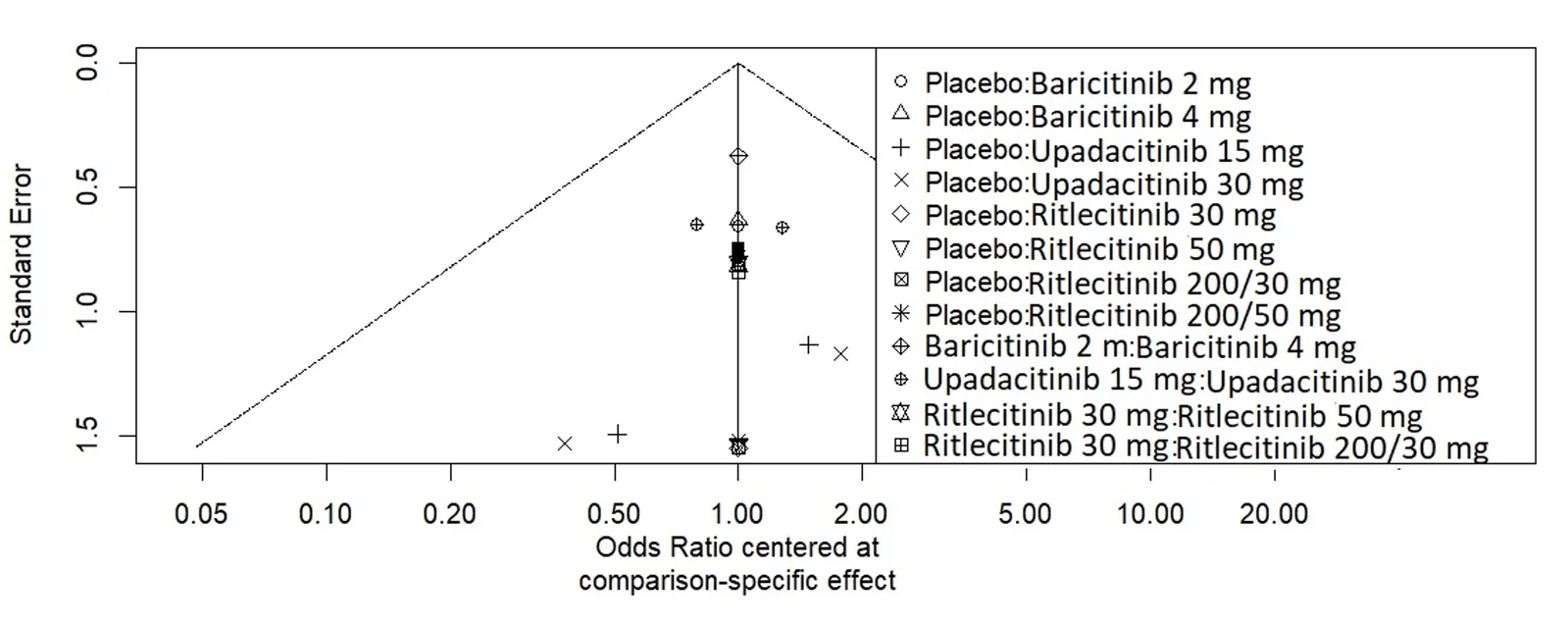
Comparison-Adjusted Funnel Plot for Publication Bias and Small-Study Effects Comparison-adjusted funnel plot from the Bayesian NMA for ritlecitinib [12], upadacitinib [31], and baricitinib [40]. No major asymmetry was observed, but interpretation is limited by few studies and sparse adolescent subgroup data. Sources: Hordinsky et al., 2023 (ritlecitinib [12]); Passeron et al., 2025 (baricitinib [40]); Mostaghimi et al., 2026 (upadacitinib [31]). NMA, network meta‑analysis.

Risk-of-Bias Assessment

The risk of bias was assessed using the Cochrane RoB 2 tool. The majority of the trials included had a low risk of bias with respect to the key domains, including randomization, deviation from the intended intervention, incomplete outcome data, and outcome measurements (Figure 7). There were some concerns in the adolescent subgroup due to a lack of detailed reporting and the exploratory nature of the study.

**Figure 7:**
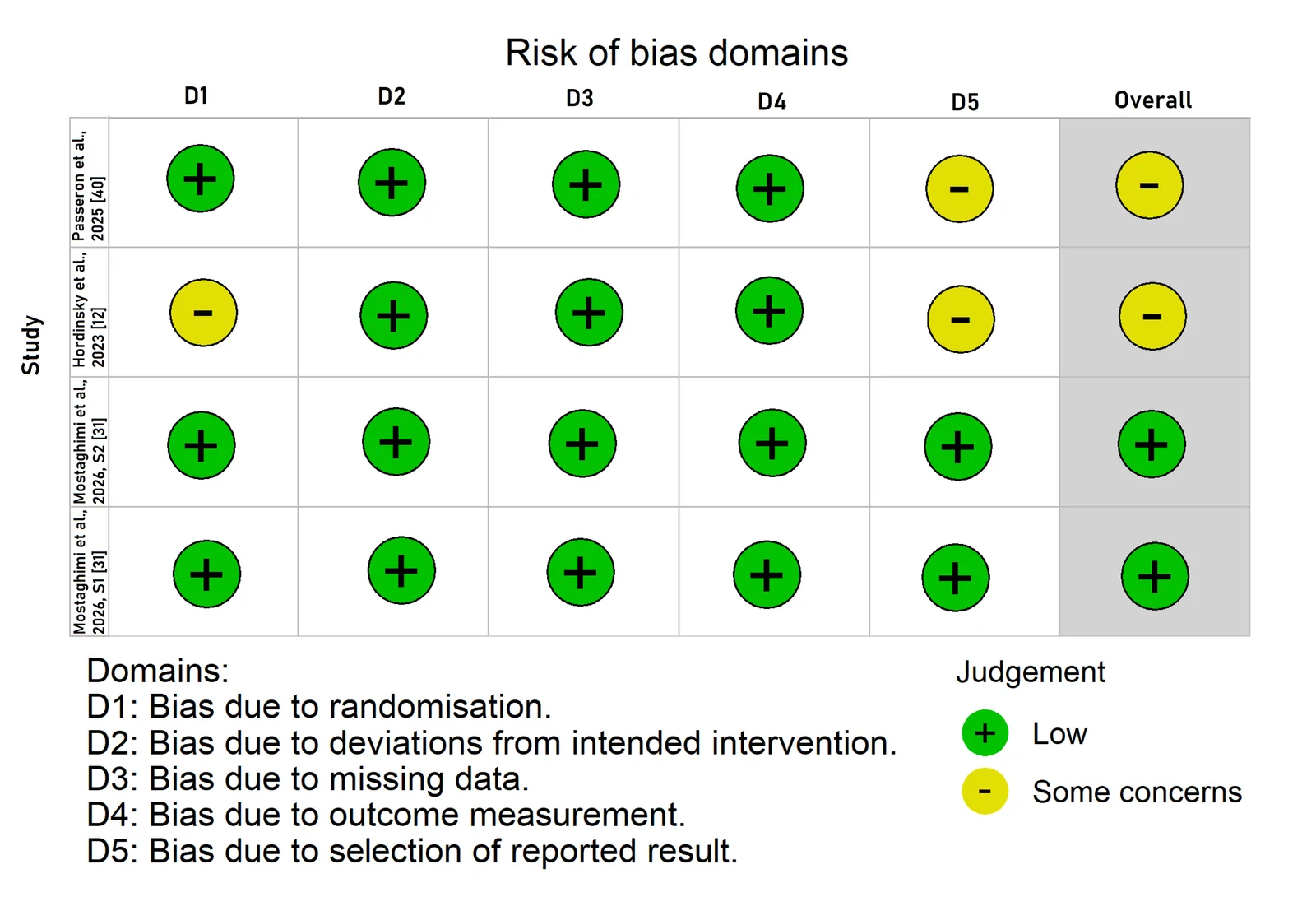
Risk-of-Bias Assessment Using the Cochrane RoB 2 Tool Risk-of-bias summary across included studies. Most domains were judged as low risk, including randomization, deviations from intended interventions, missing outcome data, and outcome measurement. Some concerns were noted in adolescent subgroup analyses because of limited reporting and an exploratory subgroup design. No study was judged to be at high overall risk.

Discussion

This Bayesian NMA evaluated the comparative efficacy of the JAK inhibitors ritlecitinib, baricitinib, and upadacitinib in adolescents with severe AA using placebo‑anchored indirect comparisons. All three agents improved the likelihood of achieving a SALT score of ≤20 relative to placebo, reinforcing the expanding role of targeted immunomodulatory therapy in this population. Among the evaluated regimens, upadacitinib 30 mg produced the most favorable and statistically stable efficacy estimates, followed by upadacitinib 15 mg, baricitinib 4 mg, and baricitinib 2 mg. In contrast, ritlecitinib regimens generated highly unstable and imprecise effect estimates, characterized by extremely large ORs and wide uncertainty ranges that limited interpretability. These features were driven by sparse adolescent subgroup data and near‑zero placebo response rates, which collectively constrained the reliability of indirect comparisons involving ritlecitinib despite its numerically elevated point estimates.

Direct Week 24 responder rates from individual trials supported these findings, with 72% to 85% of adolescents achieving a SALT score of ≤20 on upadacitinib 30 mg and 56% to 57% on upadacitinib 15 mg across two studies, compared with 32% and 17% on baricitinib 4 mg and 2 mg, and 25% on ritlecitinib 50 mg. These observed responder rates indicate that upadacitinib demonstrated the strongest efficacy profile, whereas baricitinib and ritlecitinib produced more modest and broadly similar responses. Together, these results highlight meaningful differences in therapeutic performance across available and emerging JAK inhibitors for adolescents with severe AA and underscore the importance of comparative evidence in guiding treatment selection in the absence of head‑to‑head trials.

These findings are biologically plausible given the well‑established role of the JAK‑STAT signaling pathway in AA pathogenesis [41,42]. Interferon‑gamma and other pro‑inflammatory cytokines drive immune‑mediated follicular destruction, and selective inhibition of JAK‑dependent signaling is thought to restore hair follicle immune privilege and facilitate hair regrowth. Prior pivotal clinical trials have demonstrated meaningful efficacy for both ritlecitinib and baricitinib in severe AA, leading to regulatory approval for ritlecitinib in adults and adolescents in June 2022 and for baricitinib in adults in June 2023 and adolescents in September 2026 [22-24]. Robust evidence now supports substantial clinical benefit of selective JAK1 inhibition with upadacitinib in adolescents and adults [31]. Upadacitinib has been authorized by the EMA for severe AA in both adults and adolescents and remains under review by the US FDA for this indication [26,32].

The indirect comparisons further supported a dose-response relationship for both upadacitinib and baricitinib, with higher-dose regimens demonstrating numerically greater efficacy consistent with adult and adolescents randomized controlled trial data. For ritlecitinib, although the approved dose is 50 mg for both adolescents and adults with severe AA, the present NMA included the 30 mg, 200/30 mg, and 200/50 mg regimens. This decision was justified by the comparatively narrow evidence base underlying ritlecitinib's regulatory approval, which rested on the single pivotal Phase 2b/3 study enrolling both adults and adolescents, with a relatively limited adolescent sample size. The ritlecitinib 200/30 mg and 200/50 mg treatment regimens included an initial four-week course of a daily loading dose of 200 mg, followed by 20-week courses of either 30 mg or 50 mg maintenance doses. Patients, who had received a loading dose, exhibited a better Week 24 SALT score of ≤20 response rate compared with patients treated with 50 mg and 30 mg without a loading dose [37], which suggests that high-dose therapy in the first period can increase the therapeutic efficacy. However, regardless of these numerically favorable loading‑dose results, the efficacy profile of ritlecitinib proved to be the least successful among all three tested JAK inhibitors; moreover, responder rates of ritlecitinib seemed comparable with baricitinib rates rather than approaching significantly higher rates of upadacitinib. A current clinical trial is evaluating a 100 mg ritlecitinib regimen against the approved 50-mg dose in adolescents and adults with severe AA using external and synthetic placebo controls [43], and its results may provide more definitive efficacy and safety data at higher ritlecitinib doses (NCT06873945).

The results of our study also challenge the long‑standing assumption that orally administered JAK inhibitors for severe AA achieve broadly similar efficacy, with approximately half of patients failing to reach a SALT score of ≤20 despite prolonged therapy. The Week 24 responder rates observed with upadacitinib, particularly the 72% to 85% responses achieved with the 30-mg dose across two studies, substantially exceed those previously reported for baricitinib, ritlecitinib, and deuruxolitinib in adults, as well as the baricitinib and ritlecitinib responses observed in adolescents in this analysis. These findings suggest that upadacitinib may represent a distinct efficacy tier within the JAK inhibitor class, with the potential to reduce the need for sequential intraclass switching due to inadequate response, a practice associated with added clinical and economic burden.

On the other hand, the lower responder rates achieved with baricitinib and ritlecitinib further support their placement within the efficacy tier characteristic of currently available JAK inhibitors. If confirmed in future trials, this pattern may meaningfully influence the treatment paradigm for severe AA.

Descriptive analyses of eyebrow and eyelash outcomes were broadly consistent with the primary efficacy findings. JAK inhibitor regimens generally demonstrated higher response rates than placebo, with numerically greater improvements observed with upadacitinib 30 mg and higher-dose baricitinib regimens [31,32,40]. Despite these conclusions, interpretation of eyebrow and eyelash outcomes requires caution due to inconsistent definitions of response and variable reporting across studies, which precluded formal NMA for these endpoints.

Methods of quantitative evidence synthesis such as conventional meta-analysis and NMA contribute substantially to clinical decision-making, guideline development, reimbursement considerations, and research prioritization. The validity of these approaches depends on careful evaluation of key methodological assumptions, including similarity of enrolled populations, independence of treatment comparisons, appropriate handling of effect modifiers, and coherence between direct and indirect evidence. Conventional meta-analysis requires that included studies address a comparable clinical question, use compatible effect measures, and exhibit minimal or explainable heterogeneity. NMA incorporates these same requirements and additionally assumes a connected evidence network, clearly defined intervention nodes, and the presence of both transitivity and consistency.

The above‑listed assumptions are generally satisfied in large industry‑sponsored global registration studies conducted after consultation with regulatory agencies and in accordance with internationally recognized standards of good clinical and statistical practice. These methodological strengths provide an appropriate framework for the indirect comparisons conducted in the present analysis, while also acknowledging the unique challenges posed by sparse adolescent subgroup data.

The recent regulatory approval of baricitinib for adolescents with severe AA in the US, together with the anticipated approval of upadacitinib for adolescents with severe AA, will meaningfully expand therapeutic options and provide evidence‑based alternatives to ritlecitinib, which remains the only JAK inhibitor with several years of clinical experience in this age group. Several intersecting clinical realities underscore the need for a broader therapeutic armamentarium.

A recent anonymous survey of 86 US-based board-certified dermatologists found that more than half reported a preference for attempting alternative therapies, primarily intralesional or topical corticosteroids, before initiating a JAK inhibitor [44], reflecting persistent prescriber caution around this drug class. Among the agents compared in this study, upadacitinib holds a distinct advantage for patients with comorbid atopic dermatitis, as it is the only JAK inhibitor in this analysis with an established indication for moderate to severe atopic dermatitis [30,45,46].

At a population level, more than 50% of adult and adolescent patients with severe AA fail to achieve a SALT score of ≤20 after 12 months of continuous therapy with all three currently available JAK inhibitors in the US (baricitinib, ritlecitinib, and deuruxolitinib) at the highest indicated doses, a sobering efficacy ceiling that is compounded by compelling indirect evidence of striking interindividual and intraclass variability in treatment response. These observations underscore the potential importance of upadacitinib, given its higher responder rates relative to baricitinib and ritlecitinib and its approval for patients with AA and comorbid atopic dermatitis. When initial therapy proves inadequate in efficacy or tolerability, sequential switching among available JAK inhibitors may yield incremental clinical benefit, particularly in patients who demonstrate partial response to a first agent [15-21]. Until mechanistically differentiated and potentially safer alternatives, such as rezpegaldesleukin, a first‑in‑class biologic anticipated to carry no boxed warnings or require ongoing laboratory monitoring [47], become available for the management of severe AA, including in patients with comorbid atopic dermatitis, strategic intraclass JAK inhibitor rotation will remain an important tool for optimizing outcomes in patients who do not achieve or sustain adequate responses. These observations also highlight the potential benefit of initiating therapy with upadacitinib in suitable patients, given its higher efficacy and the potential to lessen the clinical and economic burden associated with intraclass switching.

The present study has several limitations that should inform the interpretation of its findings. First, in the absence of randomized trials directly comparing JAK inhibitors in adolescents with AA, all comparative estimates were derived from indirect, placebo‑anchored NMA. As a result, these findings should be interpreted as exploratory and hypothesis‑generating rather than as definitive evidence of superiority between agents. Second, the small adolescent subgroup sample sizes across included studies contributed to statistical instability and wide CrIs for several treatment comparisons, most notably those involving ritlecitinib. Third, heterogeneity in baseline measures, including identified factors such as SALT score, duration of AA, duration of current episode, and unmeasured influences, raised considerations for the transitivity assumption. Heterogeneity in efficacy assessment timepoints and inconsistent reporting of secondary outcomes further limited the scope of comparative analyses for eyebrow and eyelash endpoints. Fourth, because clinical responses to JAK inhibitors often continue to evolve beyond the short placebo‑controlled induction windows used in most trials, this analysis cannot fully assess differences in durability, continued deepening of response, relapse dynamics, or long‑term safety, features that are highly relevant in a chronic, relapsing condition. Following discontinuation of placebo groups and randomization or conversion to active treatments for the rest of participants, there will no longer be a stable common comparator in the network, making indirect comparison during maintenance less reliable. This methodological approach also assumes that all JAK inhibitors have a similar pattern of long-term responses, which may not always be the case. Fifth, even though the surface under the cumulative ranking curve (SUCRA) criterion is extensively employed in many NMAs to interpret treatment hierarchies, its application to sparse subgroup data with unstable effect estimates risks generating misleading rankings. For this reason, SUCRA‑based hierarchy was intentionally not used in this study, and the emphasis of the analysis was instead placed on comparative effect estimates and clinical plausibility. Lastly, even though the analysis focuses on comparing the efficacy of available JAK inhibitors, a broader and more personalized evaluation is needed when selecting treatment in clinical practice because safety profiles and patient‑related factors are not incorporated into a network meta‑analytic approach. As a result, the comparative rankings presented here should be interpreted as one component of a larger evidence‑based decision process rather than a definitive guide to agent selection.

Despite these limitations, this study represents the first focused comparative evaluation of oral JAK inhibitors in adolescents with severe AA. The findings reinforce the therapeutic potential of JAK inhibition in younger patients while also highlighting the substantial evidence gaps that persist in this population. Although adequately powered head‑to‑head randomized trials remain the gold standard for comparative efficacy assessment, such trials are unlikely in this therapeutic area due to considerable financial and operational constraints. Consequently, indirect comparison methods, including Bayesian NMA, provide a valuable framework for informing relative efficacy and supporting clinical decision-making in the absence of direct evidence.

## Conclusions

This Bayesian NMA demonstrated that ritlecitinib, baricitinib, and upadacitinib improved the likelihood of achieving a SALT score of ≤20 in adolescents with severe AA relative to placebo. Across the evaluated regimens, upadacitinib 30 mg showed the most favorable and statistically stable efficacy profile, with upadacitinib 15 mg also demonstrating substantially greater efficacy than either baricitinib or ritlecitinib. Week 24 responder rates from the individual trials reinforced this hierarchy, with markedly higher responses for upadacitinib, particularly the 72% to 85% responses observed with the 30-mg dose across two studies, compared with the more modest and broadly similar responses observed with baricitinib and ritlecitinib. These findings suggest that upadacitinib may represent a distinct therapeutic tier within the JAK inhibitor class and challenge the long‑standing assumption that all oral JAK inhibitors for severe AA achieve comparable efficacy. Descriptive eyebrow and eyelash outcomes were directionally consistent with the primary findings.

These results should be interpreted with caution, as the analysis relied on indirect placebo‑anchored comparisons, small adolescent subgroup sample sizes, and heterogeneous reporting of secondary and safety outcomes. Comparisons involving ritlecitinib were particularly affected by statistical instability due to sparse data and near‑zero placebo response rates. To the authors’ knowledge, this study represents the first focused comparative evaluation of oral JAK inhibitors in adolescents with severe AA. The findings underscore the need for adequately powered, head‑to‑head randomized trials to more definitively define the optimal therapeutic approach in this population.

## References

[1] Fitzhugh MH, Hansen JG, Jabbari A, Berrebi KG (2025). Pathophysiology of alopecia areata in the pediatric patient. Pediatr Dermatol.

[2] Tan IJ, Jafferany M (2024). Psychosocial impact of alopecia areata in paediatric and adolescent populations: a systematic review. J Paediatr Child Health.

[3] Malhotra K, Madke B (2023). An updated review on current treatment of alopecia areata and newer therapeutic options. Int J Trichology.

[4] Tsianakas A, Passeron T, Magnolo N (2026). Efficacy and safety of deuruxolitinib, an oral selective Janus kinase 1/2 inhibitor, in adults with alopecia areata: results from the THRIVE-AA2 phase 3, randomized, double-blind, controlled trial. J Am Acad Dermatol.

[5] Mesinkovska N, King B, Zhang X (2024). Efficacy and safety of ritlecitinib, an oral JAK3/TEC family kinase inhibitor, in adolescent and adult patients with alopecia totalis and alopecia universalis. J Dermatol.

[6] Peeva E, Guttman-Yassky E, Banerjee A (2022). Maintenance, withdrawal, and re-treatment with ritlecitinib and brepocitinib in patients with alopecia areata in a single-blind extension of a phase 2a randomized clinical trial. J Am Acad Dermatol.

[7] King B, Ko J, Kwon O (2024). Baricitinib withdrawal and retreatment in patients with severe alopecia areata: the BRAVE-AA1 randomized clinical trial. JAMA Dermatol.

[8] King B, Senna MM, Mesinkovska NA (2024). Efficacy and safety of deuruxolitinib, an oral selective Janus kinase inhibitor, in adults with alopecia areata: results from the phase 3 randomized, controlled trial (THRIVE-AA1). J Am Acad Dermatol.

[9] Piliang M, Soung J, King B (2025). Efficacy and safety of the oral Janus kinase 3/tyrosine kinase expressed in hepatocellular carcinoma family kinase inhibitor ritlecitinib over 24 months: integrated analysis of the ALLEGRO phase IIb/III and long-term phase III clinical studies in alopecia areata. Br J Dermatol.

[10] Kwon O, Senna MM, Sinclair R (2023). Efficacy and safety of baricitinib in patients with severe alopecia areata over 52 weeks of continuous therapy in two phase III trials (BRAVE-AA1 and BRAVE-AA2). Am J Clin Dermatol.

[11] King B, Ohyama M, Kwon O (2022). Two phase 3 trials of baricitinib for alopecia areata. N Engl J Med.

[12] Hordinsky M, Hebert AA, Gooderham M (2023). Efficacy and safety of ritlecitinib in adolescents with alopecia areata: results from the ALLEGRO phase 2b/3 randomized, double-blind, placebo-controlled trial. Pediatr Dermatol.

[13] (2026). Leqselvi (deuruxolitinib) US Prescribing Information, 06/30/2026 (SUPPL-2), NDA 217900, Sun Pharmaceuticals. https://tinyurl.com/y7tw7xvf.

[14] King B, Zhang X, Harcha WG (2023). Efficacy and safety of ritlecitinib in adults and adolescents with alopecia areata: a randomised, double-blind, multicentre, phase 2b-3 trial. Lancet.

[15] Wan Z, Wang Y, Yang D (2026). Significant improvement with ivarmacitinib after suboptimal response to tofacitinib in severe alopecia areata: a case report and literature review. Front Immunol.

[16] Okazaki T, Takahashi T, Wada-Irimada M (2026). Ritlecitinib in alopecia areata: a 24-week real-world experience contrasting JAK inhibitor-naïve and JAK inhibitor-experienced patients. J Dermatol.

[17] Peterson D, Powell M, King B (2021). Less is more? Failure of one JAK inhibitor does not predict failure of another one in a patient with alopecia areata. Dermatol Ther.

[18] Kalil L, Craiglow BG, King B (2025). Successful treatment of alopecia areata with one JAK inhibitor after failure of other JAK inhibitors. JAMA Dermatol.

[19] Zhang X, Jiang Y (2024). Predictors and management of inadequate response to JAK inhibitors in alopecia areata. Am J Clin Dermatol.

[20] Silva I, Khattri S (2025). Jak inhibitor intraclass switch in alopecia areata patients: a retrospective review of cases at an academic center. Arch Dermatol Res.

[21] Martin A, Chen LC, Kreytak C (2026). Switching between Janus kinase inhibitors for treatment of alopecia areata: a multicenter retrospective review. J Am Acad Dermatol.

[22] (2026). Olumiant (baricitinib) US Prescribing Information, 06/30/2026 (SUPPL-11), NDA 207924, Eli Lilly & Co. https://tinyurl.com/muwx3jd7.

[23] (2026). Litfulo (Ritlecitinib) US Prescribing Information, 06/30/2026 (SUPPL-1), NDA 215830, Pfizer. https://tinyurl.com/3kpur4de.

[24] (2026). Olumiant (Baricitinib) Summary of Product Characteristics. https://tinyurl.com/9de3p56r.

[25] (2026). Lilly's Olumiant (baricitinib) recommended by CHMP for approval of expanded use in the European Union for adolescents with severe alopecia areata, press release, Feb 27. https://tinyurl.com/2apn3m33.

[26] (2026). AbbVie submits application to FDA for upadacitinib (RINVOQ) for adults and adolescents with severe alopecia areata. https://tinyurl.com/dhtxy8ud.

[27] Freitas E, Guttman-Yassky E, Torres T (2023). Baricitinib for the treatment of alopecia areata. Drugs.

[28] Eisman S, Sinclair R (2021). Ritlecitinib: an investigational drug for the treatment of moderate to severe alopecia areata. Expert Opin Investig Drugs.

[29] Lauletta G, Potestio L, Nappa P, Napolitano M (2026). Ritlecitinib for the management of alopecia areata: a narrative review of real-world evidence and selected post hoc analyses. Dermato.

[30] (2026). Rinvoq (upadacitinib), US Prescribing Information, 06/30/2026 (SUPPL-34), NDA 211675, Abbvie. https://tinyurl.com/3ww9jw6r.

[31] Mostaghimi A, Gooderham MJ, Lynde C (2026). Upadacitinib for severe alopecia areata in adults and adolescents: two phase 3 UP-AA randomized clinical trials. JAMA Dermatol.

[32] (2026). Upadacitinib (Rinvoq), Summary of Product Characteristics, European Medicines Agency, AbbVie Deutschland GmbH. https://tinyurl.com/mrxurshw.

[33] Mostaghimi A, Senna MM, Huynh L (2025). Treatment patterns of patients with Alopecia areata treated with FDA-approved JAK inhibitors (AB95). J Am Acad Dermatol.

[34] Liu Y, Béliveau A, Wei Y (2023). A gentle introduction to Bayesian network meta-analysis using an automated R package. Multivariate Behav Res.

[35] Hutton B, Catalá-López F, Moher D (2016). The PRISMA statement extension for systematic reviews incorporating network meta-analysis: PRISMA-NMA. Med Clin.

[36] Babul A, Ashraf S, Elmes R, Mahdavi P (2026). Epistemic barriers to buprenorphine access for adolescents with opioid use disorder. Cureus.

[37] (2026). Drug Approval Package: Litfulo, Pfizer, Inc., Approval Date: 6/23/2023. Center For Drug Evaluation and Research, Food and Drug Administration. Application Number: 215830Orig1s000 Multi-Discipline Review. https://tinyurl.com/ybanzdra.

[38] Hackenberger BK (2020). Bayesian meta-analysis now - let's do it. Croat Med J.

[39] Sterne JA, Savović J, Page MJ (2019). RoB 2: a revised tool for assessing risk of bias in randomised trials. BMJ.

[40] Passeron T, Arkin L, Ohyama M (2026). Baricitinib provides significant hair regrowth in adolescents with severe alopecia areata: 36-week efficacy and safety results from a phase 3 randomized, controlled trial. J Dtsch Dermatol Ges.

[41] Lensing M, Jabbari A (2022). An overview of JAK/STAT pathways and JAK inhibition in alopecia areata. Front Immunol.

[42] Chim I, Ghiya R, Sinclair RD, Eisman S (2024). Novel investigational drugs for alopecia areata and future perspectives. Expert Opin Investig Drugs.

[43] King B, McMichael A, Sinclair R (2025). Rationale and design of a novel, phase 3, external and synthetic placebo-controlled clinical trial of ritlecitinib 50 mg and 100 mg for alopecia areata. Dermatol Ther.

[44] Nohria A, Desai D, Lee A, Karagounis T, Shapiro J, Garshick M, Lo Sicco KI (2024). Evaluating dermatologists' knowledge of and attitudes toward Janus kinase inhibitor therapy for the treatment of alopecia areata. J Am Acad Dermatol.

[45] Ly S, Manjaly P, Kamal K (2023). Comorbid conditions associated with alopecia areata: a systematic review and meta-analysis. Am J Clin Dermatol.

[46] Lebwohl MG, Lain EL, McMichael A, Senna MM, Mesinkovska NA (2026). Alopecia areata: a clinical review of the changing landscape with Janus kinase inhibitors. JAAD Rev.

[47] (2026). Nektar Therapeutics: REZOLVE-A phase 2b study of rezpegaldesleukin establishes proof-of-concept in patients with severe-to-very-severe alopecia areata. https://tinyurl.com/22vsr3z8.

